# Speeding the growth of primary mental health prevention

**DOI:** 10.1186/s13584-015-0008-9

**Published:** 2015-03-13

**Authors:** Lawrence S Wissow

**Affiliations:** Johns Hopkins School of Public Health, 703 Hampton House, 624 North Broadway, Baltimore, MD 21205 USA

## Abstract

While there is a strong case for primary prevention of mental health problems, relatively little mental health scholarship has been devoted to it in the last decade. Efforts to accelerate prevention scholarship could potentially benefit from strengthening pathways for interdisciplinary research; developing new training and working models for mental health professionals; developing a common language for public, policy, and scientific discussion of prevention; learning how to measure the common outcomes of heterogeneous interventions tailored to diverse communities.

## Commentary

In 1974, Canada’s “Lalonde Report” (formally “A new perspective on the health of Canadians”) was among the first policy statements by a major government to recognize that health care involved more than the treatment of acute and chronic illness [1]. It extended the government’s responsibility to include both the prevention of illness and the promotion of well-being. In 1984, the US established its Preventive Services Task Force and in 1996 the UK its National Screening Committee. Prevention of many somatic illnesses, and promotion of somatic health, have become cornerstones of both public health and clinical medical services. Parallel programs for mental health, while present and spreading, lag behind in scope [2,3]. A recent European consensus statement listed new approaches to positive mental health, well-being, and protective factors as its lead priority for public mental health research [4]. In this context, it is not surprising that Nakash and colleagues [5] found that less than 10% of Israeli mental health scholarship over the last decade seems to have been devoted to prevention.

Nakash and colleagues make an eloquent case for the need for primary prevention and they set out come concrete steps within academics and clinical services. We can set out some possible challenges and opportunities to consider in taking these steps.

### Who champions prevention?

First, there is the question of where prevention has a home and who will be its champions. One has only to look at the range of disciplines represented by the strategies for prevention suggested by the WHO’s Comprehensive Mental Health Action Plan [6]: human rights, stigma, economic policies and inequalities [7,8], promotion of nurturing parent–child relationships [9], school-readiness and subsequent education [10], and healthy and satisfying work conditions. To this list one could add work in architecture, landscape, and urban design meant to reduce stress, promote social interaction, and influence lifestyle choices related to mental and physical health [11]. This multi- and trans-disciplinary nature of prevention challenges how most current academic work is organized, perhaps with the exception of intentionally chaotic schools of public health. Grant-makers look for focused projects, trainees are encouraged to learn their own discipline, and mentors warn that too much collaboration early in one’s career could slow attaining status as an independent scholar. Institutions have yet to fully organize around research and implementation frameworks that address a full socio-ecologic model [12], to which many would add an explicit business or financial plan [13].

### Clinical roles

Primary mental health prevention can be equally challenging for clinicians’ traditional roles. Mental health clinicians working with individuals who already have an identified problem or risk can be alert for the needs of other family members [14]. As Nakash and colleagues suggest, mental health clinicians can also collaborate and consult with medical providers who treat individuals with somatic issues that put them at risk of mental health problems [15], or who provide health maintenance and anticipatory guidance [16]. They can also collaborate with schools, law enforcement, or child protection programs. But even though these collaborations and extensions of care beyond the identified patient have long been recognized as important, they receive less emphasis in training programs and frequently are not factored into funding schemes. There are few incentives for mental health clinicians not to fill their agendas with already-ill individuals in need of treatment, and few incentives for non-mental health clinicians to expand their scope of work to include early intervention for mental health problems [17]. One hope for the future is that new structures in health care will be able to improve integration between medical, social, and public health sectors. For example, the Health and Wellbeing Boards that recently began operation in larger UK cities [18], will be able to advocate for and influence financing of more flexible roles for specialists and generalist health care providers, as well as synergistic cross-sector efforts at both an individual patient and community level.

### In search of a common model and language

Perhaps one of the biggest barriers to work in primary mental health prevention is finding common language and models from which to work, and thus some agreement on how interventions across sectors can be fashioned into a coherent whole. There may be some agreement that prevention needs to avert not just mental illness but also sub-threshold levels of distress that impair function, and that doing so involves the promotion of mental wellness – people’s ability to feel good about themselves, get along with others, and feel up to meeting everyday challenges [19]. But debates and advocacy for particular models can further fragment prevention efforts, setting up competition for scare resources or government attention.

One area in which prevention policy advocates may clash involves timing. Clearly prevention needs to start early in life, or even before conception. There are periods, mostly in early childhood but extending into young adulthood, when brains are most sensitive to positive and negative environmental effects [20,21]. Threat and chronic physical or psychological adversity early in life may weaken or under-develop brain regions involved in learning, memory, and the ability to self-regulate that are critical to life-long success [22], in favor of regions important to survival in adverse circumstances, those that promote aggressive responses and increase monitoring for danger [21]. Inversely, there is evidence that safe, stimulating, and nutritionally-adequate environments in early childhood can promote the acquisition of both the cognitive and non-cognitive skills that both promote social success and extend the individual’s capacity for healthy adaptation to life’s certain adversities [10].

However, human brains have life-long capacities to develop new abilities and tailor the profile of an individual’s faculties in response to environmental demands. Early gains can be lost without ongoing support in middle childhood [21]; new work supports the impact on brain structure of nurturing parent behaviors in adolescence as well as in early childhood [9]. In fact, primary preventive interventions are possible across the lifespan, either to promote healthy aging or in response to medical or social adversities [23].

Another tension with policy implications is whether the factors that promote either positive mental health or resilience in the face of stressors should be seen as traits inherent to the individual or characteristics of the individual’s environment [24]. Clinical approaches tend to weigh more heavily on the individual’s own traits, but various forms of social capital and structural aspects of the individual’s surroundings may play an even more important role. Even when clinicians can formulate a patient’s needs using a more ecologic model, they may lack a way to translate recognition into concrete suggestions or links to related services.

Models can also be developed that have great appeal to policy makers but that are potentially stigmatizing or off-putting for individuals who may ultimately be the target of preventive services. As an example, currently in North America, the concept of “adverse childhood experiences” [25] leading to “toxic stress” and damage to both “the genome and the brain” [26] has gained much traction with policy makers and the general public [27]. However, in clinical practice, some at-risk families worry that revealing their adversities could result in child protection interventions, and find the concept that they have been poisoned by stress both stigmatizing and discouraging. Sometimes the most effective models in terms of spreading interventions do not mention the explicit goals, or embed goals within related programs that already have wide acceptance [23]. If much of preventive work addresses change in individual behavior and social norms, then the models and communication strategies involved in promoting that change require careful articulation.

### No “single best strategy”

Finally, a huge challenge to prevention may be that it is yet another example of the need to think globally but act very locally. Not only is the design of complex, multi-faceted prevention programs dependent on the unique human resources available in any given community [28], but there may be no “single best strategy” for helping individuals deal with adversity [20]. At a clinical level there may be some broadly-applicable interventions that serve to promote brain plasticity [29] – physical exercise, positive social interactions are two examples – but even these might not be practical everywhere, and the interventions that take advantage of the plasticity to build or shift the balance of abilities may need to be individualized. Thus, another challenge to researchers, clinicians, and service planners is how to develop a diverse set of interventions, to be skilled but nimble in their use, and to find a way to track and sum up quality and outcomes across very different programs and settings.

### Speeding up the process

Balas and colleagues [30] have helped sensitize the medical community to the delays, often measured in decades, from the time new knowledge appears until in reaches everyday use. The figures they cite about screening for diabetic retinopathy are sobering – 12 years from the initial “landmark” publication until inclusion in clinical guidelines, and then at 20 years evidence that less than half of patients with diabetes were receiving guideline-consistent care. Nakash and colleagues’ review [5] of the Israeli literature is a strong call for expansion of prevention research and training. Several steps - strengthening pathways for interdisciplinary research; developing new training and working models for mental health professionals; developing a common language for public, policy, and scientific discussion of prevention; learning how to measure the common outcomes of heterogeneous interventions -- could potentially speed the movement of new prevention science into clinical and public health programs.
